# Fate of a Famous Health Resort

**Published:** 1896-03

**Authors:** 


### Fate of a Famous Health Resort.

Forty years ago Mentone was a healthy village in France, where lived peasantry happy in their farms and their superb physical state, conditioned by the climate. It was discovered that the region was a most healing climate for consumptives, and it became the Mecca for the unfortunates of Europe so stricken. The inhabitants abandoned their farms to wait upon the strangers. The strong, healthy women forsook their dairies and became the washerwomen of the consumptives' clothes. No precautions were taken; the disease was not then understood as now, the theory of the tubercle bacillus not having been discovered. The place to-day is bacillus ridden, a pest-hole, death itself. The hitherto strong inhabitants are emaciated, a coughing, bleeding people, filled with the germs of consumption. The soil and the air are both contaminated with them. It is no longer a resort. The same fate, it is believed, awaits many other similar health resorts unless active means are taken to destroy all germs. This will be a most difficult task, because consumptives themselves, as a rule, are not thoughtful of the danger they spread, or of the rights of others. They should bear in mind that if all others had been careful they, too, might have escaped.—*Journal of Hygiene.*

